# Proton Therapy and Src Family Kinase Inhibitor Combined Treatments on U87 Human Glioblastoma Multiforme Cell Line

**DOI:** 10.3390/ijms20194745

**Published:** 2019-09-24

**Authors:** Francesco P Cammarata, Filippo Torrisi, Giusi I Forte, Luigi Minafra, Valentina Bravatà, Pietro Pisciotta, Gaetano Savoca, Marco Calvaruso, Giada Petringa, Giuseppe A. P. Cirrone, Anna L Fallacara, Laura Maccari, Maurizio Botta, Silvia Schenone, Rosalba Parenti, Giacomo Cuttone, Giorgio Russo

**Affiliations:** 1 Institute of Molecular Bioimaging and Physiology, National Research Council, IBFM-CNR, 90015 Cefalù, Italy; francesco.cammarata@ibfm.cnr.it (F.P.C.); giusi.forte@ibfm.cnr.it (G.I.F.); valentina.bravata@ibfm.cnr.it (V.B.); savoca.gaetano@gmail.com (G.S.); marco.calvaruso@ibfm.cnr.it (M.C.); giorgio.russo@ibfm.cnr.it (G.R.); 2 National Institute for Nuclear Physics, Laboratori Nazionali del Sud, INFN-LNS, 95123 Catania, Italy; filippo.torrisi@unict.it (F.T.); pietro.pisciotta@lns.infn.it (P.P.); giada.petringa@lns.infn.it (G.P.); pablo.cirrone@lns.infn.it (G.A.P.C.); cuttone@lns.infn.it (G.C.); 3 Department of Biomedical and Biotechnological Sciences (BIOMETEC), University of Catania, 95123 Catania, Italy; parenti@unict.it; 4 Departments of Physics and Astronomy, University of Catania, 95123 Catania, Italy; 5 Lead Discovery Siena s.r.l. (LDS), 53100 Siena, Italy; al.fallacara@gmail.com (A.L.F.); l.maccari@leaddiscoverysiena.it (L.M.); maurizio.botta@unisi.it (M.B.); 6 Department of Biotechnology, Chemistry and Pharmacy, Università degli Studi di Siena, 53100 Siena, Italy; 7 Department of Pharmacy, Università degli Studi di Genova, 16126 Genova, Italy; schenone@difar.unige.it

**Keywords:** glioblastoma multiforme, proton therapy, combined treatments, gene signatures

## Abstract

Glioblastoma Multiforme (GBM) is the most common of malignant gliomas in adults with an exiguous life expectancy. Standard treatments are not curative and the resistance to both chemotherapy and conventional radiotherapy (RT) plans is the main cause of GBM care failures. Proton therapy (PT) shows a ballistic precision and a higher dose conformity than conventional RT. In this study we investigated the radiosensitive effects of a new targeted compound, SRC inhibitor, named Si306, in combination with PT on the U87 glioblastoma cell line. Clonogenic survival assay, dose modifying factor calculation and linear-quadratic model were performed to evaluate radiosensitizing effects mediated by combination of the Si306 with PT. Gene expression profiling by microarray was also conducted after PT treatments alone or combined, to identify gene signatures as biomarkers of response to treatments. Our results indicate that the Si306 compound exhibits a radiosensitizing action on the U87 cells causing a synergic cytotoxic effect with PT. In addition, microarray data confirm the SRC role as the main Si306 target and highlights new genes modulated by the combined action of Si306 and PT. We suggest, the Si306 as a new candidate to treat GBM in combination with PT, overcoming resistance to conventional treatments.

## 1. Introduction

Glioblastoma multiforme (GBM) is a central nervous system tumor classified as grade IV of high-grade malignant gliomas (HGG), according to the World Health Organization (WHO) guidelines [1]. GBM belongs to the group of diffuse astrocytic and oligodendroglial tumor, joining oligodendrocytomas, ependymomas, and mixed gliomas, under the glioma classification [2]. According to the ASTRO guidelines statements, the current standard care for GBM is surgical resection to the feasible extent, followed by conventional radiotherapy (RT) of 60 Gy delivered by fractions of 2 Gy, up to seven weeks. Moreover, chemotherapy is concurrent to RT with daily temozolomide (TMZ) administration [3,4,5]. These treatment modalities are not currently curative and the resistance to both chemotherapy and RT plans is the main cause of GBM care failures (the median survival time is 14.6 months) [6]. Moreover, the percentage of relapses and side effects post TMZ and RT treatments is more than 90% [7]. More precisely, even if the application of TMZ has significantly improved clinical GBM outcomes, cases of drug resistance related to the activity of the enzyme methyl guanine methyl transferase (MGMT) have been observed [8]. The hypermethylation of its promoter, is indeed associated with a better survival rate in patients receiving TMZ with or without RT [9]. In addition, the dose release onto healthy brain tissue or surrounding organs at risk during irradiation may, substantially, contribute to late tissue toxicities, such as radionecrosis and neurocognitive dysfunction, because of their limited dose tolerance.

In recent years, different dose fractionation schedules have been improved to have a better prognosis, avoiding the large side effect even in case of focal re-irradiation of recurrences. In this scenario, proton therapy (PT) could be used as a successful strategy for GBM treatment, being able to regulate the balance between tumor control and the normal tissue tolerance [10,11,12,13,14]. In particular, when heavy particles cross the tissues, they deposit a minimal radiation dose on their track to the tumor. The depth-dose distribution, described by the Bragg peak trend, gradually increases as a function of the depth. So, the so-called spread-out Bragg peak (SOBP) lead to a complete irradiation of the target volume and a more conformal dose distribution, sparing the surrounding healthy tissues from damage [15,16]. This specific dose distribution curve represents a key topic for GBM tumor treatments in which the sparing of healthy tissue is a key factor for the patient’s quality of life. Therefore, there is a robust scientific rationale motivating the need to enlarge studies that guide towards new clinical trials for PT combined with targeted therapy rather than conventional RT with photons or electrons [17,18].

Today, in the context of personalized medicine, prognostic and predictive molecular biomarkers are useful to select cancer therapeutic planning [19,20]. A critical point in RT success is the prediction of cancer radiosensitivity. At the molecular level, the idea that genes may behave as biomarkers of a disease response represents the base for the development of gene signatures, to predict response to cancer radiation treatments [21]. Several genes have been shown to be responsive to radiation exposure and thanks to the use of high-throughput technologies, such as gene expression profiling (GEP) by microarray, radiosensitivity assays have been developed with gene signatures predicting radioresponse in many cancer types, including GBM [22]. However, the response to radiation is highly cell-line dependent and some specific genes and pathways may be linked both to tumor subtypes and dose delivered [23,24,25].

Actually, few published studies have evaluated the effectiveness of radiosensitizing agents combined with PT in GBM and none of them consider genes and response pathways induced by RT. Most studies have demonstrated that different genetic pathways and molecular features can provide reliable prognostic biomarkers, overlooking the treatment responses and predictive outcomes. However, according to WHO guidelines, IDH1/IDH2 gene status distinguishes a more radioresistant tumor type (primary GBM, IDH-wild type) from a more sensitive one (secondary GBM, *IDH-mutant*). IDH mutation is correlated with epigenetic modifications of the MGMT gene and assumes a prognostic value together with other biomarkers such as, the presence of LOH 10q, epidermal growth factor receptor (EGFR) amplification, p16^INK4a^ deletion, TP53 mutation, PTEN mutation, and the co-deletion of 1p/19q [26,27,28].

Based on this evidence, a large group of molecularly targeted agents have been designed, but none of them seem to overcome tumor radioresistance [29]. Previous studies support an involvement of the SRC-family protein kinases in the irradiation induction of radioresistance mechanisms. SRC protein is a non-receptor tyrosine kinase that interacts with many intracellular proteins involved in GBM carcinogenesis and progression. In addition, in vitro and in vivo studies confirmed the correlation between SRC activity and GBM carcinogenesis. [30].

In this work we analyzed the GEP on the U87 MG human glioblastoma cell line after treatment with PT alone or in combination with a new targeted compound, named Si306 (Lead Discovery Siena, Siena, Italy), inhibiting SRC proteins. The Si306 molecule is a new TKI, chosen among the family of pyrazolo[3,4-d] pyrimidines, that exhibited the ability to specifically bind the ATP site of SRC protein, making it inactive. Furthermore, previous in vitro and in vivo studies have shown that the Si306 determines a significant reduction of the β-PDGFR active phosphorylated form and a greater loss of the migratory ability in GBM cells stimulated by Epidermal Growth Factor (EGF). In addition, the antiproliferative effect of Si306 has been tested in association with conventional RT treatments both in vitro and in vivo [31].

Here, in order to clarify the Si306 activity in GBM cells exposed to PT, we firstly evaluated radiosensitive effects of different amounts of the Si306 compound on the U87 cell line in combination with PT exposed at the doses of 1, 2, 3, 4, 10, and 21 Gy. Clonogenic assay and dose modifying factor (DMF) calculations were performed. We also analyzed the U87 cell radiosensitivity by applying the radiobiological linear-quadratic (LQ) model and calculated the α, β, and $\frac{\alpha }{\beta }$ ratio, commonly used to predict radiosensitivity of normal and tumor cells [32].

In addition, at molecular level we selected 2 and 10 Gy of proton radiation doses combined with the Si306 to evaluate GEP induced responses, by using whole genome cDNA microarray. We described networks and specific gene signatures of response to both treatments, highlighting for the first time, the cell pathways induced by Si306.

## 2. Results

### 2.1. IC50 Determination

In order to evaluate cytotoxicity ability of Si306 in term of concentration that determined the 50% of growth inhibition (IC_50_), U87 cells were incubated with Si306 at increasing concentrations of 0.1, 1.0, 10, and 100 μM for 24, 48, and 72 h under normal cell culture conditions. Cell numbers and viability were evaluated and the IC_50_ values calculated at each time points (Table 1).

### 2.2. Cell Radiosensitization Following Combined Treatments with Protons and Si306

**Figure 1:**
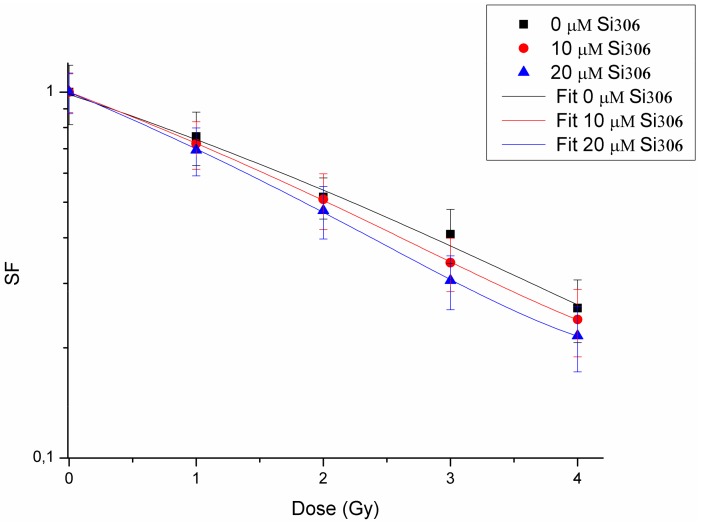
Cell survival curves of U87 cells. Cells treated with protons only (black line), protons plus 10 µM of Si306 (red line), and protons plus 20 µM of Si306 (blue line).

To evaluate the radiosensitizing ability of Si306 compound, we investigated the combined effects of this molecule on U87 cells exposed to different proton doses (1, 2, 3, 4, 10, and 21 Gy). Surviving fraction values, obtained by clonogenic assay, after irradiation with protons alone or after pretreatment with 10 and 20 µM Si306, are shown in Table 2. These surviving fraction (SF) values were plotted to obtain dose-response curves with the exception of the 10 Gy and 21 Gy doses because of the lack of LQ model validity at high doses (Figure 1). We then calculated the DMF, which represents the relative reduction of dose to be delivered following a combined treatment with Si306 to get the isoeffect of SF = 0.5 compared to radiation treatment without modification. The DMF values were 1.09 (10 µM of Si306) and 1.21 (20 µM of Si306), showing a radiosensitive effect at both concentrations (Table 3).

### 2.3. LQ Model

We calculated LQ parameters α and β of U87 cells, which provided information about the intrinsic cell radiosensitivity. Together with $\frac{\alpha }{\beta }$ ratio they have a pivotal role for a reliable estimation of radiation response, although most of the studies reported a large heterogeneity in LQ parameters and limited data is published about PT [33,34]. The U87 fitted survival curve, generated after only protons administration, gives us the values of 0.292 Gy^-1^ for α and of 0.010 Gy^−2^ for β, that result in an $\frac{\alpha }{\beta }$ ratio of 28.6 Gy (Table 4).

The higher $\frac{\alpha }{\beta }$ ratio showed, when the Si306 is added, especially at higher concentrations, determines a more linear cell survival as reasonably expected and demonstrates the molecule radiosensitivity role. Moreover, the shape variations at the origin of survival curves are linked with the DMF values. Other points are evident for the relationship between the LQ parameters and survival curve. Si306 affects substantially the linear component (α), whereas the quadratic component (β) is slightly decreased at higher concentrations. These results can be interpreted according to the LQ model, in which the cell death is lead, in our case, to the greater accumulation of lethal lesions. The use of Si306, both at concentrations of 10 and 20 μM, combined with PT contributes to sensitize GBM cells to protons exposure with an increase in cell killing.

### 2.4. Gene Expression Profiles (GEP) Experiments

As a second aim of this work, here we have reported GEP data obtained applying a Two-Color cDNA Microarray-Based Gene Expression Analysis (Agilent technologies) on U87 cells exposed to PT, with or without 10 µM Si306 compound. Comparative differential gene-expression analysis revealed that multiple deregulated genes (DEG) were significantly altered, by 2-fold or greater according to the specific experimental configuration reported as follows.

In addition, as described by several authors and also by our group [35,36], we have studied GEP lists, using PubMatrix, a tool for multiplex literature mining, in order to confirm our assumptions and to test their involvement in selected queries, radiation related, to draw assumptions described in the “Discussion” section. In this way, lists of terms, such as gene names, were assigned to a genetic, biological, or clinical relevance in a flexible systematic fashion in order to confirm our hypothesis, highlighting the involvement of known and lesser known genes able to drive cell radiation responses (Table S1).

#### 2.4.1. GEP Induced by Proton Irradiation in U87 Glioblastoma Cells

Firstly, we analyzed the gene expression changes uniquely induced by protons irradiation with 2 and 10 Gy of IR doses. It should be remembered that 2 Gy is the daily dose delivered in fractionated RT treatments, so it is a dose of clinical interest, while 10 Gy represents a high dose of interest for comparisons with high-dose GEP studies of our research group [36].

In particular, U87 cell line treated with 2 Gy changed the expression levels of 936 genes (215 down regulated and 721 up regulated). On the other hand, 1018 DEGs were selected in U87 cells treated with 10 Gy and, among these, 251 were down regulated while 767 up regulated (Table 5).

Deregulated transcripts obtained were grouped by using the DAVID tool [37,38] according to pathway analysis and the top-five molecular pathways selected are reported in Table 6. The analysis on DEGs induced by PT treatment with 2 Gy revealed the involvement of a set of factors controlling cellular processes, such as Hippo signaling pathway, cAMP signaling pathway, antigen processing and presentation, Wnt signaling pathway, and cell adhesion molecules (CAMs).

**Figure 2:**
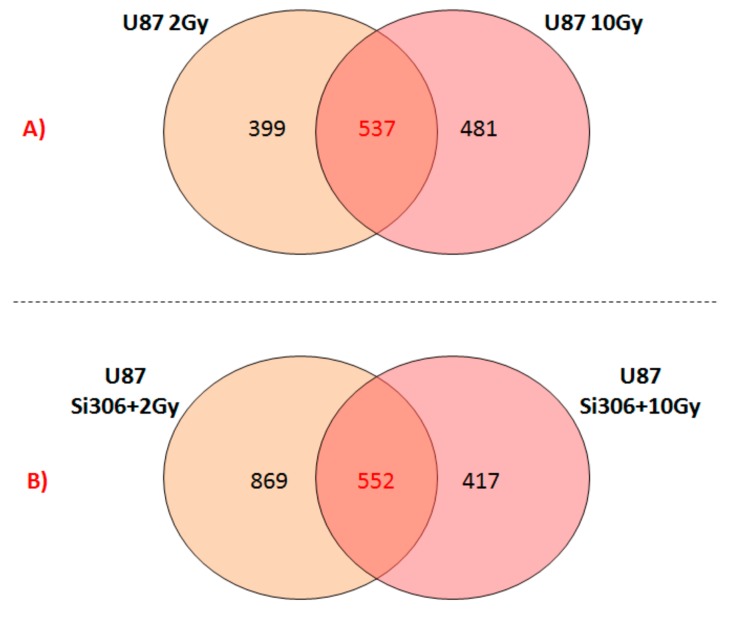
Venn diagrams showing the number of unique and shared differentially expressed genes after exposure to: (**A**) PT and (**B**) Si306 + PT combined treatments.

On the other hand, U87 glioblastoma cells exposed to 10 Gy of proton irradiation activate specific cell pathways as displayed in Table 6: PI3K-Akt signaling pathway, p53 signaling pathway, proteoglycans in cancer, Hippo signaling pathway, and cAMP signaling pathway. Finally, the GEP lists were analyzed by Venn diagrams in order to identify the overlapping deregulated genes (537 DEGs), between the two configurations of 2 and 10 Gy assayed (Figure 2A). Some genes were specifically deregulated following the dose provided, showing a dose-dependent transcriptional response. Moreover, cells respond to radiation treatment also in a common manner with activation of common genes and pathways, as displayed in Table 6 and listed as follows: Hippo signaling pathway; cAMP signaling pathway; proteoglycans in cancer; neuroactive ligand-receptor interaction; and antigen processing and presentation. Except for the neuroactive ligand-receptor interaction pathway, formed overall by molecules driving neuronal cell signaling, the involvement of these cellular processes in U87 cells proton exposed, has described above.

#### 2.4.2. GEP Induced by Si306 and Proton Combined Treatments in U87 Glioblastoma Cells

In a second step, we have evaluated the effect on GEPs after a combined administration of 10 µM Si306 compound and PT using the doses of 2 and 10 Gy, hereafter named as follows: U87 Si306 + 2 Gy and U87 Si306 + 10 Gy, which were analyzed in comparison to the respective samples treated with PT alone (U87 2 Gy and U87 10 Gy). We selected a large amount of deregulated genes, caused by the Si306 compound addition to PT treatment: 1419 DEGs (563 down and 856 up regulated) in U87 Si306 + 2 Gy, while 969 DEGs (353 down and 616 up regulated) changed their expression levels in U87 Si306 + 10 Gy (Table 5). Thus, also for these experimental configurations, up and down regulated transcripts were grouped according to their involvement in specific biological pathways using DAVID tool [38]. The top-five statistically relevant molecular pathways of deregulated gene datasets are reported in Table 7. In particular, the Si306 + 2 Gy combined treatments deregulated the expression levels of genes controlling: Phagosome, antigen processing and presentation, cell adhesion molecules, inflammatory disease, and calcium signaling pathway.

Some of the above described pathways were also deregulated in U87 cells exposed to Si306 + 10 Gy and following reported and listed in Table 7: Proteoglycans in cancer, leukocyte transendothelial migration, phagosome, cell adhesion molecules, and autoimmune disease. Three out of the five pathways selected in U87 Si306 + 10 Gy (proteoglycans in cancer, phagosome, and cell adhesion molecules), were also deregulated in the other configurations analyzed, underling once again their interesting role in U87 cells response to radiation and/or to the Si306 molecule.

Finally, the Venn diagram shown in Figure 2B displays 552 deregulated common genes between the two configurations: U87 Si306 + 2 Gy and U87 Si306 + 10 Gy. The top-five statistically relevant pathways selected by DAVID tool using the 552 common gene list, are displayed in Table 7, and following listed: Autoimmune disease, antigen processing and presentation, proteoglycans in cancer, apoptosis, and inflammatory bowel disease.

## 3. Discussion

The first purpose of this study was to evaluate the radiosensitizing effects mediated by combination of the new compound, the Si306 targeting SRC proteins, with PT on the U87 human glioblastoma cell line. The IC_50_ evaluation showed that this cell line is sensitive to treatment with the Si306 compound. Based on the IC_50_ values, we tested the radiosensitizing effect of Si306, used at concentrations of 10 and 20 μM, in combination with proton irradiation at increasing doses of 1,2, 3, 4, 10, and 21 Gy, in order to generate dose/response curves for the dose configurations tested.

The radiosensitizing effect was evaluated by calculating the DMF, obtained at the SF of 50%, in order to highlight the combined treatment capacity of enhancing tumor cells killing in respect of irradiation only [39]. Our data show that pretreatment with Si306 at both concentrations leads to a synergic cytotoxic effect with PT on the U87 cell line, further suggesting this compound as a new possible candidate to treat GBM in combination with PT. Indeed, the possibility to use drug/IR combined treatments, permits to increase the tumor control probability (TCP) even for radioresistant tumors, such as GBM. In addition, we also analyzed the U87 cell radiosensitivity by applying the radiobiological LQ model calculating the α, β parameters, and $\frac{\alpha }{\beta }$ ratio, which predict the radiosensitivity of normal and tumor cells [32]. The LQ model is considered to be the best-fitting model to describe cell survival and, therefore, is of great interest in radiation oncology to highlight the link existing between the $\frac{\alpha }{\beta }$ ratio and the following RT-induced tissue reactions [34,40,41]. The $\frac{\alpha }{\beta }$ ratio obtained on U87 cell line is in line with the $\frac{\alpha }{\beta }$ ratio calculated for a population of glioma cells reported by Barazzuol et al., who used a mathematical model to extract radiobiological information from clinical GBM patients data [42]. In addition, our results showed a higher $\frac{\alpha }{\beta }$ ratio by using combined treatments of Si306 and protons. Therefore, we speculate that the clinical effect of using combined treatments of PT/Si306 administration, with an optimized Si306 pharmacological quantity for the patients, could be translated into the possibility of modifying the PT schedule treatment. Thus, all of this gains an efficacy in TCP, by using a more tolerable fractionated PT treatment plan and a reduced total dose delivered to the tumor [43,44].

As a second aim of this work, we carried out a transcriptomic study in order to define gene signatures as biomarkers of treatment response. GEP by whole genome cDNA microarray was firstly performed to analyze the gene expression changes uniquely induced by proton irradiation with 2 and 10 Gy of IR doses, which represent two clinical doses of interest and also for comparison with high-dose GEP studies of our research group [23,24,36,45].

In particular, the treatment of U87 with 2 Gy revealed that a large number of genes were deregulated and involved in the regulation of specific cellular processes (Table 6). One of the activated pathways was the Hippo signaling pathway, an emerging growth control and tumor suppressor pathway that regulates cell proliferation and stem cell functions; the hyperactivation of its downstream effectors (such as TAZ protein, up regulated in U87 2 Gy with a fold change of 1,89) contributes to the development of cancer including GBM, suggesting that pharmacological inhibition of these factors may be an effective anticancer strategy [46,47]. In turn, in GBM cells Yang et al. recently reported that the Hippo transducer TAZ promotes cell proliferation and tumor formation through the EGFR pathway [48]. In addition, Hippo and Wnt signaling, up regulated in U87 2 Gy cells, reciprocally regulate each other’s activity through a variety of mechanisms that needs to be better clarified in GBM cells [49]. As known, Wnt/β-catenin signaling plays important roles in maintaining the stemness of cancer stem cells in various cancer types and in promoting cellular invasiveness. Multimodality in vivo and in vitro studies revealed a key role of Wnt activation in GBM radiation resistance. In turn, literature data report a pivotal role of the Wnt/β-catenin signaling pathway in IR-induced invasion of U87 GBM cells, indicating that β-catenin is a potential therapeutic target for overcoming evasive radioresistance [50,51].

In U87 2 Gy the involvement of cAMP signaling pathway was also observed. Existing evidence suggests that intracellular cAMP level and signaling may affect the survival of cancer cells, including resistant cancer cells to standard chemotherapeutic drugs. Suppression of the cAMP pathway is a common feature across different cancers including GBM. [52,53]. In addition, IR is known to be able to activate the transcription of genes, through the presence of cAMP responsive elements (CREs) in their promoters, in order to guide cell response and survival after radiation exposure [54].

Moreover, the activation of antigen processing and presentation pathway after proton exposure with dose of 2 Gy in GBM cells is sustained by an up regulation of genes belonging to the human leukocyte antigen (HLA) class family (probably activated by β-catenin), factors involved in antigen presentation. As reported by Ghosh et al., HLA genes increasing level, often caused by a hypoxic tumor microenvironment, is associated with evasion of immune responses in cancer cells [55]. Finally, an overall activation of several cell adhesion molecules was highlighted in U87 2 Gy cells, involved in the activation of inflammation process and in the regulation of cancer invasiveness.

On the other hand, U87 cells exposed to 10 Gy of proton irradiation activate specific cell pathways, including the phosphatidylinositol-3-kinase (PI3K)-protein kinase B (Akt) signaling pathway (Table 6). As known, the PI3K/AKT pathway is commonly activated in cancer initiation and progression, including GBM, as it regulates different processes, such as proliferation, apoptosis, and migration [56], therefore representing a key target for cancer therapeutics. Moreover, the activation of TP53 pathway was observed in U87 10 Gy and driven by TP53 gene that was significantly altered by 1.77-fold. As described, TP53, exerts a crucial role following IR-induced DNA damage because it is able to cause cell cycle arrest, DNA repair, and apoptosis processes. Moreover, the influence of TP53 status on DNA damage repair after cell irradiation has been studied in several malignancies and also reported by our group in breast cancer cells after a high dose of electron irradiation [45,57]. Finally, in U87 10 Gy, an activation of proteoglycan signaling was observed. Proteoglycans are known to have many roles in tumor progression and are the main extracellular matrix (ECM) components of normal brain tissue, playing an important role in brain development; an overproduction of different molecules of this family were found in GBM cells [58,59].

Interestingly, in U87 10 Gy Hippo and cAMP signaling pathways were activated, as above described in U87 2 Gy configuration, underling once again the important role of these processes in GBM cells after proton exposure.

In a second step, we evaluated the GEPs induced by Si306 molecule in U87 cells irradiated with 2 and 10 Gy of proton doses and we selected a large number of deregulated genes, grouped according to their involvement in specific biological pathways (Table 7). In particular, in U87 Si306 + 2 Gy combined treatments a deregulated expression level of genes controlling phagosome was observed.

In GBM an intensive autophagic activity regulated by several signaling pathways was described [60]. As recently reported by Yasui et al., an altered autophagic flux was described in GBM cell lines exposed to 10 Gy of γ-rays. Our data also confirms this trend after proton exposure. These altered fluxes represent a useful biomarker of metabolic stress induced by IR and provide a metabolic context for radiation sensitization [61]. Here the Si306 radiosensitization effect seems to act by stressing this molecular mechanism. In addition, in U87 Si306 + 2 Gy configuration the involvement of antigen processing and presentation and cell adhesion molecules pathways were observed, similarly to that shown in U87 cells proton treated with only 2 Gy. Therefore, the Si306 treatment seems to cause an overall down regulation of HLA molecules (up regulated in U87 2 Gy), suggesting the activation of immune surveillance escaping mechanism induced by Si306 [55,62].

The latest two pathways deregulated in U87 Si306 + 2 Gy were linked to inflammation and calcium signaling. As known, the inflammation process is often activated in cell exposed to radiation, affecting cell fate by the activation of key transcription factors (TFs), such as NF-KB and STATs (i.e., STAT1 and STAT3) [63]. Interestingly, the combined Si306 + 2 Gy treatment induced a down regulation of STAT1 and STAT3 proteins. Thus, we speculate that this inhibition could promote radiation sensitivity decreasing angiogenesis and cell survival as hypothesized in other malignancies by several authors [64,65]. Indeed, a number of studies confirm that selective inhibitors of these proinflammatory pathways driven by STAT TFs, could be combined to conventional radiation or chemotherapy to increase their effectiveness [66,67].

On the other hand, the combined treatment with Si306 and 2 Gy PT seem to affect survival/death balance by modulating the intracellular calcium levels, a mechanism known to be involved in regulating IR-induced cell cycle arrest, apoptosis, and chromatin structure modifications [45,68,69].

Some of these pathways were also deregulated in U87 cells exposed to Si306 + 10 Gy, such as: Proteoglycans in cancer, leukocyte transendothelial migration, phagosome, cell adhesion molecules, and autoimmune disease. Three of the five pathways (proteoglycans in cancer, phagosome, and cell adhesion molecules), were also deregulated in the other configurations analyzed, suggesting once again their important role in U87 cells in response to radiation and/or to Si306 molecule. The other two selected pathways in U87 Si306 + 10 Gy (i.e., leukocyte transendothelial migration and autoimmune disease), highlight the involvement of a complex immunological response induced by IR, as known from the literature, and by the Si306 compound addition, as observed in this study.

Finally, we reported the number of overlapping deregulated genes between the two configurations of the combined treatments, such as U87 Si306 + 2 Gy and U87 Si306 + 10 Gy (Figure 2B). The top-five statistically relevant pathways selected and displayed in Table 7, were previously described.

**Figure 3:**
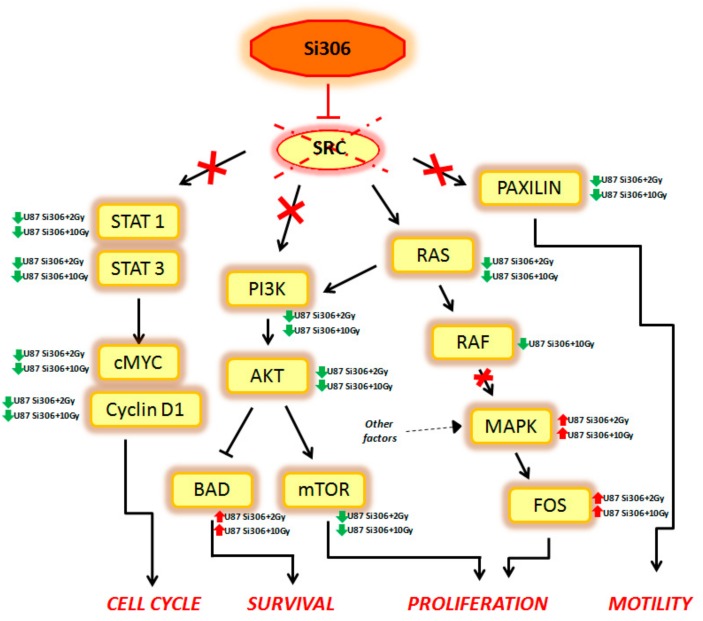
The figure displays the main targets of Si306 compound observed. The arrows define an activation and the T bars the inhibition. Red arrows define gene upregulation and green arrows gene downregulation.

Summarizing, our GEP results show that combined treatments on U87 cells can activate multiple signal transduction pathways described, to our knowledge, for the first time, to be new targets of Si306. Finally, considering that the main target of Si306 is the tyrosine kinase SRC, we analyzed the known cellular target downstream to this transducer, in order to better clarify its role as molecular radiosensitizing. Thus, we observed that the combined treatment Si306 + protons (with 2 and 10 Gy) in U87 cells, is able to inhibit several signal transduction pathways, normally regulated by SRC as shown in Figure 3.

In particular, the STAT1, STAT3, c-MYC, and Cyclin D1 genes, which are able to control the cell cycle, were downregulated in our analysis. Cell survival was negatively regulated by the downstream PI3K, AKT, and mTOR downregulation and by the BAD upregulation. In addition, Si306 is able to cause a partial inhibition of cell proliferation, downregulating RAS and RAF gene expression. However, the MAPK and FOS genes were not targets of Si306, so these factors (up regulated in our data), were probably activated by other cellular pathways. Finally, Si306 is also able to negatively regulate cell motility, through the downregulation of the paxilin gene.

These data confirm the SRC role as a main target of Si306 compound and highlight the transcriptional events occurring downstream of SRC inhibition by the combined treatments. The SRC blockage observed after Si306 and PT combined treatments seems to increase the single treatments effectiveness, thus promoting a radiosensitizing effect.

Today, very little data is available regarding the combination of molecularly targeted drugs and PT. Indeed, many studies debate about chemotherapeutic agents combined with high-linear energy transfer (LET) particle beams or protons for GBM treatment, overlooking the clinical perspective of target therapy [70,71].

The results obtained from this work have highlighted the radiosensitizing capacity of the Si306 targeted compound on U87 GBM cell line, acting in tandem with PT. Taking into account previously in vivo pharmacokinetic data, demonstrating that Si306 was able to reach the brain, overcoming the hurdle represented by the blood–brain barrier [31], this compound can be considered a new candidate for combined treatments of GBM. In addition, our GEP results confirm the important role of SRC as the main Si306 target and highlight new genes and pathways modulated by the combined action of Si306 and PT, which can be further explored as new radiosensitizing therapeutic targets in GBM.

## 4. Materials and Methods

### 4.1. Proton Irradiation Configuration and Cell Irradiation

The proton beam irradiation was performed at the CATANA (Centro di Adroterapia ed Applicazioni Nucleari Avanzate) facility of INFN-LNS (Catania, Italy) [72], using 62 MeV of proton beams accelerated by a cyclotron superconducting. The beamline is composed of several passive elements optimized for the clinical application: Scattering foils to spread the beam laterally, collimators to define beam profile in accordance to the tumor shape, and monitoring chambers to measure the dose delivered [73]. In order to irradiate the entire 25 cm^2^ (T25) standard tissue culture flasks, a motorized system for biological samples irradiation was used. Radiochromic film detectors were adopted to check the delivered dose and the lateral dose distribution during each irradiation. The dosimetric system was calibrated under reference conditions according to the International Atomic Energy Agency Technical Reports Series No. 398 “Absorbed Dose Determination in External Beam Radiotherapy” [74].

For combined treatments with Si306, U87 irradiations were carried out using six dose values of 1, 2, 3, 4, 10, and 21 Gy. Cell irradiations were conducted placing the cell at the middle spread-out Bragg peak, to simulate a clinical condition, with a dose rate of 15 Gy/min.

### 4.2. Cell Culture and IC50 Determination

In vitro experiments were carried out using the U87 MG human glioblastoma cell line. Cells were purchased from European Collection of Authenticated Cell Cultures (ECACC, Public Health England, Porton Down Salisbury, UK) and cultured as previously described [31]. Cells were maintained in an exponentially growing culture condition in an incubator at 37 °C in a humidified atmosphere (95% air and 5% CO_2_) and were routinely sub-cultured in T25 standard tissue culture flasks.

To calculate IC_50_ (drug concentration that determined the 50% of growth inhibition), 2.5 × 10^4^ U87 cells were plated in 12-well plates and incubated with Si306 dissolved in DMSO at increasing concentrations (0.1, 1.0, 10, and 100 μM) for 24, 48, and 72 h under normal cell culture conditions. Cell numbers and viability were evaluated using Z2 Coulter Counter (Beckman Coulter, Indianapolis, United States). IC_50_ was calculated by GraphPad Prism 6.0 software (GraphPad Software, San Diego, CA, USA) using the best fitting sigmoid curve.

### 4.3. Clonogenic Survival Assay

Forty-eight hours before irradiations U87 cells were seeded in T25 flasks and the day after were incubated with the concentrations of 10 and 20 µM of Si306, chosen on the base of IC_50_ results, for 24 h prior to radiation treatment. Cells were irradiated at subconfluence. Combined effects of Si306 and protons were evaluated by clonogenic survival assay, performed as previously described [45,57]. Briefly, after irradiation, U87 cells were detached, counted by hemocytometer and seeded into a six-well plate in triplicate at a density of 50–2000 cells per well, by plating an increasing cell quantity according to the dose delivered raising, in order to assay the SF. Colonies were allowed to grow under normal cell culture conditions for two weeks and then were fixed with 50% methanol and stained 0.5% crystal violet (both from Sigma-Aldrich, St. Louis, MO, USA). Colonies with more than 50 cells were counted manually under Olympus CK30 phase-contrast microscope (Olympus, Tokyo, Japan) and also automatically with a computer-assisted methodology [75]. The calculation of SFs in U87 cells irradiated with protons and pre-treated with Si306 were determined taking into consideration the plating efficiency (PE) for all treatment modalities based on three independent experiments.

### 4.4. The Linear-Quadratic Model

The linear-quadratic model, introduced by Kellerer and Rossi in the 1970s [32], is the most widely used model in RT, in which a lethal event is supposed to be caused by one hit due to one particle track (the linear component αD) or two particle tracks (the quadratic component $\beta D^{2}$).

The clonogenic survival data were analyzed by means of non-linear regression, which utilizes a multi-parameter equation for curves, whose form is: S(D)/S(0)=e ^(-αD-βD^2)^, so we get α[Gy^-1^] e β[Gy^-2^] with their own standard deviation.

### 4.5. Dose Modifying Factor Calculation

The parameter dose modifying factor was calculated in order to evaluate synergistic effect of protons combined with Si306 compound. This value, as the best measure of treatment effectiveness, was calculated at the SF of 50% and represents the relative dose of irradiation required to obtain the isoeffect of SF = 0.5 with radiation treatment alone in respect of combined treatments with a defined concentration of Si306 [39].

The SF data versus dose were plotted with the reported quadratic equation: $y=a+bx+cx^{2}$ where y is ln(SF) and x is the dose, considering the positive solution. The experimental samples (pretreated with 10 or 20 µM of Si306 and proton irradiated) were normalized to coefficient a of the previous equation in order to start the survival curves from the same origin. The results were achieved with the software OriginPro 8.

The SF values take into account two errors: The first was derived from the equation $y=a+bx+cx^{2}$ and was calculated using error propagation; the second was derived from ratio normalization, but negligible compared to first one.

### 4.6. Whole Genome cDNA Microarray Expression Analysis

To study molecular pathways and cell networks activated at transcriptional level in U87 cells exposed to PT, with or without Si306 compound, we performed gene expression experiments by cDNA microarray. In particular, in this work we analyzed GEP of the following configurations: (i) U87 2 Gy versus U87 untreated cells; (ii) U87 10 Gy versus U87 untreated cells; (iii) U87 2 Gy + 10 µM Si306 versus U87 2 Gy; and iv) U87 10 Gy + 10 µM Si306 versus U87 10 Gy.

RNA extraction and analyses were performed as previously described [45,57]. Microarray experiments conducted by using the protocol Two-Color Microarray-Based Gene Expression Analysis (Agilent Technologies, Santa Clara, CA, USA), statistical analyzes carried out with GeneSpring GX 10.0.2 software (Agilent Technologies), and pathway analysis conducted by using DAVID database, were performed as previously described [76].

The data showed in this work were deposited in the Gene Expression Omnibus (GEO) database (NCBI) [38] and are available by using the GEO Series accession number: GSE127989.

## 5. Conclusions

The data here described, supported by DMF calculation and LQ model analyses, indicate that a new compound, the Si306 targeting SRC protein, exerts a radiosensitizing action on the U87 MG cell line causing a synergic cytotoxic effect when combined with PT. This compound can be considered a new possible candidate to treat GBM in combination with PT. In addition, we provide for the first time a description of GEPs induced by Si306 and PT combined treatments, highlighting the modulated cellular networks and confirming the important role of SRC as the main target of the compound. Taking together our encouraging data suggest the use of Si306 compound in targeted therapies in tandem with PT, to obtain a more successful treatment modality in GBM disease.

## Figures and Tables

**Table 1:** IC_50_ values calculated after 24, 48, and 72 h of treatment with Si306 on U87 glioblastoma cell line.

IC50	IC50	IC50
24 h	48 h	72 h
17.3 μM	6.8 μM	1.98 μM

**Table 2:** Surviving fraction (SF) values of U87 cells after irradiation with only protons and after combined treatments with 10 and 20 µM of Si306.

Dose (Gy)	SF (Only Protons)	SF (Protons + 10 µM Si306)	SF (Protons + 20 µM Si306)
0	1.000 ± 0.185	1.000 ± 0.121	1.000 ± 0.127
1	0.756 ± 0.126	0.722 ± 0.107	0.694 ± 0.104
2	0.516 ± 0.066	0.509 ± 0.088	0.474 ± 0.078
3	0.409 ± 0.069	0.342 ± 0.057	0.305 ± 0.051
4	0.257 ± 0.050	0.239 ± 0.050	0.216 ± 0.044
10	0.109 ± 0.022	0.072 ± 0.018	0.064 ± 0.018
21	0.056 ± 0.015	0.039 ± 0.009	0.035 ± 0.012

**Table 3:** Dose modifying factor (DMF) values calculated as isoeffective dose at surviving fraction of 0.5.

Treatment	SF 50% (Gy)	DMF
Protons	2.22	1
Protons + 10 μM Si306	2.03	1.09
Protons + 20 μM Si306	1.84	1.21

**Table 4:** Values of the α and β parameters estimated by fitting the cell survival to the linear-quadratic (LQ) model.

Treatment	α (Gy-1)	β (Gy-2)	α/β (Gy)
Proton	0.292 ± 0.036	0.010 ± 0.003	28.6
Proton + 10 μM Si306	0.322 ± 0.011	0.010 ± 0.003	32.2
Proton + 20 μM Si306	0.372 ± 0.018	0.004 ± 0.001	93.0

**Table 5:** Number of genes significantly deregulated by 2-fold or greater in all the configuration modalities assayed in this work.

Configuration	Number of Genes	Down	Up
U87 2 Gy versus U87 n.t	936	215	721
U87 10 Gy versus U87 n.t	1018	767	251
U87 +Si306 + 2 Gy versus U87 2 Gy	1419	563	856
U87 + Si306 + 10 Gy versus U87 10 Gy	969	353	616

**Table 6:** Top-five statistically relevant pathways activated in U87 cells exposed to proton therapy (PT).

		Pathway Name	Genes Count	%	*p* Value	Genes
2 Gy	1	Hippo signaling pathway	19	0.016	0.000255	*WNT5A*, *DVL3*, *WNT10A*, *NF2*, *FZD3*, *TCF7L2*, *LLGL1*, *LATS2*, *TP73*, *DVL1*, *CTNNB1*, *PPP1CA*, *CCND3*, *CSNK1E*, *CCND2*, *DLG4*, *PARD6G*, *WNT6*, *BMP8B*
	2	cAMP signaling pathway	18	0.015	0.012333	*FXYD2*, *HCN2*, *VAV3*, *MAP2K2*, *GRIN1*, *GRIN2A*, *ATP1A4*, *VIPR2*, *ADORA1*, *AKT1*, *ATP2B2*, *PPP1CA*, *GRIN2D*, *ABCC4*, *CALML6*, *HCN4*, *PIK3R3*, *HTR1D*
	3	Antigen processing and presentation	9	0.007	0.026474	*CIITA*, *KLRC2*, *HLA-A*, *NFYC*, *HLA-C*, *HSPA1A*, *HLA-B*, *CTSB*, *HLA-E*
	4	Wnt signaling pathway	13	0.011	0.029905	*WNT5A*, *WNT10A*, *DVL3*, *FZD3*, *TCF7L2*, *DVL1*, *CTNNB1*, *SFRP1*, *CCND3*, *CSNK1E*, *CCND2*, *NFATC2*, *WNT6*
	5	Cell adhesion molecules (CAMs)	13	0.011	0.036193	*PVR*, *LRRC4*, *ITGAL*, *CD276*, *HLA-A*, *HLA-C*, *HLA-B*, *HLA-E*, *SDC4*, *NRCAM*, *SDC1*, *ITGB8*, *CLDN1*
10 Gy	1	PI3K-Akt signaling pathway	31	0.025	0.000968	*CSH1*, *PHLPP1*, *FGF7*, *PGF*, *KITLG*, *RPS6KB2*, *BCL2L1*, *GNG8*, *AKT1*, *COL6A5*, *COL6A3*, *TEK*, *COL6A2*, *COL6A1*, *PRKAA2*, *INSR*, *GHR*, *AKT2*, *FN1*, *TNXB*, *PKN2*, *HSP90B1*, *CDKN1A*, *EIF4E*, *CCND3*, *GNB2*, *CCND2*, *ITGA5*, *VEGFA*, *MDM2*, *LAMC2*
	2	p53 signaling pathway	11	0.008	0.001175	*PPM1D*, *CDKN1A*, *CCND3*, *CCND2*, *BBC3*, *BAX*, *MDM2*, *FAS*, *GADD45B*, *SESN1*, *TP73*
	3	Proteoglycans in cancer	21	0.017	0.001320	*ERBB2*, *RPS6KB2*, *IGF2*, *FLNC*, *FLNA*, *PXN*, *CTNNB1*, *AKT1*, *WNT7B*, *SDC1*, *PPP1CA*, *CDKN1A*, *MAPK12*, *ITGA5*, *VEGFA*, *MDM2*, *FAS*, *MSN*, *WNT6*, *FN1*, *AKT2*
	4	Hippo signaling pathway	15	0.012	0.012836	*NF2*, *TEAD1*, *TCF7L2*, *LATS2*, *TP73*, *DVL1*, *CTNNB1*, *WNT7B*, *PPP1CA*, *CCND3*, *BBC3*, *CCND2*, *PARD6G*, *WNT6*, *BMP8B*
	5	cAMP signaling pathway	18	0.014	0.013410	*FXYD2*, *HCN2*, *VAV3*, *GRIN1*, *HTR4*, *ATP1A4*, *VIPR2*, *ADORA1*, *AKT1*, *ATP2B2*, *FOS*, *PPP1CA*, *SSTR1*, *GRIN2D*, *HTR6*, *ABCC4*, *HCN4*, *AKT2*
Common between 2 and 10 Gy	1	Hippo signaling pathway	12	0.018	0.001636	*PPP1CA*, *CCND3*, *NF2*, *CCND2*, *PARD6G*, *WNT6*, *TCF7L2*, *BMP8B*, *LATS2*, *TP73*, *CTNNB1*, *DVL1*
	2	cAMP signaling pathway	13	0.019	0.004726	*HCN2*, *FXYD2*, *VAV3*, *GRIN1*, *ATP1A4*, *VIPR2*, *ADORA1*, *AKT1*, *ATP2B2*, *PPP1CA*, *GRIN2D*, *ABCC4*, *HCN4*
	3	Proteoglycans in cancer	12	0.018	0.013466	*AKT1*, *PPP1CA*, *SDC1*, *MAPK12*, *ERBB2*, *IGF2*, *MSN*, *FLNC*, *WNT6*, *FLNA*, *PXN*, *CTNNB1*
	4	Neuroactive ligand-receptor interaction	14	0.021	0.025160	*CSH1*, *PRLHR*, *GRIN1*, *DRD4*, *ADORA1*, *VIPR2*, *NTSR2*, *CRHR2*, *CHRM3*, *GALR3*, *GRIN2D*, *GALR2*, *UTS2R*, *CHRNA1*
	5	Antigen processing and presentation	6	0.009	0.044750	*NFYC*, *HLA-C*, *HSPA1A*, *HLA-B*, *CTSB*, *HLA-E*

**Table 7:** Top-five Statistically relevant pathways activated in U87 cells pretreated with Si306 and exposed to PT.

		Pathway Name	Genes Count	%	*p* Value	Genes
2 Gy	1	Phagosome	23	0.013	0.00014	*HLA-DQB1*, *NOS1*, *HLA-DRB1*, *MRC2*, *HLA-A*, *HLA-C*, *HLA-B*, *ITGB3*, *SFTPA1*, *HLA-E*, *CLEC4M*, *FCAR*, *CD209*, *COMP*, *TUBAL3*, *HLA-DPA1*, *SCARB1*, *HLA-DPB1*, *HLA-DOA*, *TUBB1*, *ATP6V0D2*, *TUBB4A*, *HLA-DRA*
	2	Antigen processing and presentation	15	0.009	0.00017	*CIITA*, *HLA-DQB1*, *HLA-DRB1*, *HLA-A*, *HLA-C*, *HSPA1A*, *HLA-B*, *HLA-E*, *CD74*, *KIR3DL3*, *HSPA6*, *HLA-DPA1*, *HLA-DPB1*, *HLA-DOA*, *HLA-DRA*
	3	Cell adhesion molecules (CAMs)	21	0.012	0.00036	*PVR*, *HLA-DQB1*, *HLA-DRB1*, *SELL*, *CLDN5*, *HLA-A*, *NLGN1*, *CTLA4*, *HLA-C*, *HLA-B*, *HLA-E*, *CLDN15*, *ALCAM*, *NCAM2*, *SDC1*, *CD2*, *MADCAM1*, *HLA-DPA1*, *HLA-DPB1*, *HLA-DOA*, *HLA-DRA*
	4	Inflammatory bowel disease (IBD)	13	0.007	0.00041	*HLA-DQB1*, *HLA-DRB1*, *TBX21*, *RORC*, *STAT1*, *STAT3*, *IL12RB2*, *IL17A*, *IL1B*, *HLA-DPA1*, *HLA-DPB1*, *HLA-DOA*, *HLA-DRA*
	5	Calcium signaling pathway	19	0.011	0.02473	*ORAI2*, *PTGER1*, *NOS1*, *ERBB4*, *TNNC1*, *ERBB3*, *ERBB2*, *STIM2*, *OXTR*, *EDNRA*, *ATP2B2*, *P2RX1*, *CHRM3*, *LTB4R2*, *GRPR*, *CHRNA7*, *CALML6*, *PLCB2*, *CACNA1B*
10 Gy	1	Proteoglycans in cancer	22	0.019	0.000094	*NANOG*, *ERBB4*, *ROCK2*, *HCLS1*, *ERBB2*, *FASLG*, *IGF2*, *FZD3*, *HGF*, *DCN*, *ITGB3*, *MMP2*, *PXN*, *KDR*, *CTNNB1*, *SMO*, *MAPK13*, *HPSE*, *PLCG2*, *HSPB2*, *PRKACA*, *TWIST1*
	2	Leukocyte transendothelial migration	12	0.010	0.01064	*ITGAL*, *ROCK2*, *MAPK13*, *PLCG2*, *CLDN5*, *CTNND1*, *MYLPF*, *RAPGEF3*, *JAM2*, *MMP2*, *PXN*, *CTNNB1*
	3	Phagosome	14	0.012	0.01214	*HLA-DQB1*, *HLA-DRB1*, *SFTPA1*, *ITGB3*, *COLEC11*, *TUBA8*, *CD36*, *FCGR2B*, *PIKFYVE*, *TUBAL3*, *HLA-DPA1*, *HLA-DPB1*, *TUBB1*, *TUBB4A*
	4	Cell adhesion molecules (CAMs)	12	0.010	0.03671	*HLA-DQB1*, *NRCAM*, *ITGAL*, *HLA-DRB1*, *CLDN5*, *NLGN1*, *CTLA4*, *HLA-DPA1*, *HLA-DPB1*, *JAM2*, *SELE*, *PDCD1LG2*
	5	Autoimmune disease	6	0.005	0.06648	*HLA-DQB1*, *HLA-DRB1*, *CTLA4*, *FASLG*, *HLA-DPA1*, *HLA-DPB1*
Common between 2 and 10 Gy	1	Autoimmune disease	6	0.009	0.00768	*HLA-DQB1*, *HLA-DRB1*, *CTLA4*, *FASLG*, *HLA-DPA1*, *HLA-DPB1*
	2	Antigen processing and presentation	6	0.009	0.03468	*HLA-DQB1*, *HLA-DRB1*, *KIR3DL3*, *HLA-DPA1*, *HLA-DPB1*, *CD74*
	3	Proteoglycans in cancer	10	0.015	0.04961	*ERBB4*, *MAPK13*, *ERBB2*, *FASLG*, *FZD3*, *HGF*, *ITGB3*, *MMP2*, *KDR*, *TWIST1*
	4	Apoptosis	5	0.007	0.06011	*DFFB*, *CYCS*, *CASP8*, *FASLG*, *IL3RA*
	5	Inflammatory bowel disease (IBD)	5	0.007	0.06604	*HLA-DQB1*, *HLA-DRB1*, *TBX21*, *HLA-DPA1*, *HLA-DPB1*

## Supplementary Materials

Supplementary materials can be found at https://www.mdpi.com/1422-0067/20/19/4745/s1.

## References

[1] Hanif F., Muzaffar K., Perveen K., Malhi S.M., Simjee S.U. (2017). Glioblastoma multiforme: A review of its epidemiology and pathogenesis through clinical presentation and treatment. Asian Pac. J. Cancer Prev..

[2] Louis D.N., Perry A., Reifenberger G., Von Deimling A., Figarella-Branger D., Cavenee W.K., Ellison D.W., Ohgaki H., Wiestler O.D., Kleihues P. (2016). The 2016 World Health Organization classification of tumors of the central nervous system: A summary. Acta Neuropathol..

[3] Urbańska K., Sokołowska J., Szmidt M., Sysa P. (2014). Glioblastoma multiforme—An overview. Contemp. Oncol..

[4] Khosla D. (2016). Concurrent therapy to enhance radiotherapeutic outcomes in glioblastoma. Ann. Transl. Med..

[5] Cabrera A.R., Kirkpatrick J.P., Fiveash J.B., Shih H.A., Koay E.J., Lutz S., Reardon D.A., Petit J., Chao S.T., Brown P.D. (2016). Radiation therapy for glioblastoma: An astro evidence-based clinical practice guideline. Pract. Radiat. Oncol..

[6] Stupp R., Mason W.P., van den Bent M.J., Weller M., Fisher B., Taphoorn M.J., Belanger K., Brandes A.A., Marosi C., Bogdahn U. (2005). Radiotherapy plus concomitant and adjuvant temozolomide for glioblastoma. N. Engl. J. Med..

[7] Sherriff J., Tamangani J., Senthil L., Cruickshank G., Spooner D., Jones B., Brookes C., Sanghera P. (2013). Patterns of relapse in glioblastoma multiforme following concomitant chemoradiotherapy with temozolomide. Br. J. Radiol..

[8] Lee S.Y. (2016). Temozolomide resistance in glioblastoma multiforme. Genes Dis..

[9] Rivera A.L., Pelloski C.E., Gilbert M.R., Colman H., De La Cruz C., Sulman E.P., Aldape K.D., Bekele B.N. (2010). MGMT promoter methylation is predictive of response to radiotherapy and prognostic in the absence of adjuvant alkylating chemotherapy for glioblastoma. Neuro-oncology.

[10] Fitzek M.M., Thornton A.F., Rabinov J.D., Lev M.H., Pardo F.S., Munzenrider J.E., Hedley-Whyte E.T., Okunieff P., Braun I., Hochberg F.H. (1999). Accelerated fractionated proton/photon irradiation to 90 cobalt gray equivalent for glioblastoma multiforme: Results of a phase II prospective trial. J. Neurosurg..

[11] Mizumoto M., Tsuboi K., Igaki H., Yamamoto T., Takano S., Oshiro Y., Sugahara S., Hayashi U., Hashii H., Kanemoto A. (2010). Phase I/II trial of hyperfractionated concomitant boost proton Radiotherapy for supratentorial glioblastoma multiforme. Int. J. Radiat. Oncol. Biol. Phys..

[12] Mizumoto M., Yamamoto T., Ishikawa E., Matsuda M., Takano S., Ishikawa H., Tsuboi K., Okmura T., Sakurai H., Matsumura A. (2016). Proton beam therapy with concurrent chemotherapy for glioblastoma multiforme: Comparison of nimustine hydrochloride and temozolomide. J. Neurooncol..

[13] Matsuda M., Kohzuki H., Ishikawa E., Yamamoto T., Akutsu H., Takano S., Matsumura A., Mizumoto M., Tsuboi K. (2018). Prognostic analysis of patients who underwent gross total resection of newly diagnosed glioblastoma. J. Clin. Neurosci..

[14] Petr J., Platzek I., Hofheinz F., Mutsaerts H.J., Asllani I., van Osch M.J., Jentsch C., Maus J., Troost E.G.C., Baumann M. (2018). Effects on brain tissue volume and perfusion. Radiother. Oncol..

[15] Baumann M., Krause M., Overgaard J., Debus J., Bentzen S.M., Daartz J., Bortfeld T., Richter C., Zips D. (2016). Radiation oncology in the era of precision medicine. Nat. Rev. Cancer.

[16] Tommasino F., Durante M. (2015). Proton Radiobiology. Cancers.

[17] Combs S., Schmid T., Vaupel P., Multhoff G. (2016). Stress response leading to resistance in glioblastoma-The need for innovative radiotherapy (iRT) Concepts. Cancers.

[18] Tusuboi K. (2018). Advantages and Limitations in the Use of Combination Therapies with Charged Particle Radiation Therapy. Int. J. Part. Ther..

[19] Hirst D.G., Robson T. (2010). Molecular biology: The key to personalised treatment in radiation oncology?. Br. J. Radiol..

[20] Dalton W.S., Friend S.H. (2006). Cancer biomarkers—An invitation to the table. Science.

[21] Speers C., Pierce L.J. (2017). Molecular signatures of radiation response in breast cancer: Towards personalized decision-making in radiation treatment. Int. J. Breast Cancer..

[22] Meng J., Li P., Zhang Q., Yang Z., Fu S. (2014). A radiosensitivity gene signature in predicting glioma prognostic via EMT pathway. Oncotarget.

[23] Bravatà V., Cammarata F.P., Minafra L., Pisciotta P., Scazzone C., Manti L., Savoca G., Petringa G., Cirrone G.A.P., Cuttone G. (2019). Proton-irradiated breast cells: molecular points of view. J. Radiat. Res..

[24] Minafra L., Bravatà V., Cammarata F.P., Russo G., Gilardi M.C., Forte G.I. (2018). Radiation gene-expression signatures in primary breast cancer cells. Anticancer Res..

[25] Bravatà V., Cava C., Minafra L., Cammarata F., Russo G., Gilardi M., Forte G., Castiglioni I. (2018). Radiation-induced gene expression changes in high and low grade breast cancer cell types. Int. J. Mol. Sci..

[26] Yang P., Zhang W., Wang Y., Peng X., Chen B., Qiu X., Li W., Li G., Li S., Wu C. (2015). IDH mutation and MGMT promoter methylation in glioblastoma: Results of a prospective registry. Oncotarget.

[27] Szopa W., Burley T.A., Kramer-Marek G., Kaspera W. (2017). Diagnostic and therapeutic biomarkers in glioblastoma: Current status and future perspectives. BioMed Res. Int..

[28] Karsy M., Neil J.A., Guan J., Mahan M.A., Colman H., Jensen R.L. (2015). A practical review of prognostic correlations of molecular biomarkers in glioblastoma. Neurosurg. Focus.

[29] Sottili M., Gerini C., Desideri I., Loi M., Livi L., Mangoni M. (2016). Tumor microenvironment, Hypoxia, and Stem Cell-Related Radiation Resistance. Radiobiology of Glioblastoma: Recent Advances and Related Pathobiology; Current Clinical Pathology.

[30] Ahluwalia M., De Groot J., Liu W., Gladson C.L. (2010). Targeting SRC in glioblastoma tumors and brain metastases: Rationale and preclinical studies. Cancer Lett..

[31] Calgani A., Vignaroli G., Zamperini C., Coniglio F., Festuccia C., Di Cesare E., Botta M., Gravina G.L., Mattei C., Vitale F. (2016). Suppression of SRC Signaling Is Effective in Reducing Synergy between Glioblastoma and Stromal Cells. Mol. Cancer Ther..

[32] Chapman J.D. (2014). Can the two mechanisms of tumor cell killing by radiation be exploited for therapeutic gain?. J. Radiat. Res..

[33] Van Leeuwen C.M., Oei A.L., Crezee J., Bel A., Franken N.A.P., Stalpers L.J.A., Kok H.P. (2018). The alfa and beta of tumours: A review of parameters of the linear-quadratic model, derived from clinical radiotherapy studies. Radiat. Oncol..

[34] Bentzen S.M., Joiner M.C. (2009). The linear-quadratic approach in clinical practice. Basic Clin. Radiobiol..

[35] Becker K.G., Hosack D.A., Dennis G., Lempicki R.A., Bright T.J., Cheadle C., Engel J. (2003). PubMatrix: A tool for multiplex literature mining. BMC Bioinform..

[36] Bravatà V., Minafra L., Cammarata F.P., Pisciotta P., Lamia D., Marchese V., Petringa G., Manti L., Cirrone G.A., Gilardi M.C. (2018). Gene expression profiling of breast cancer cell lines treated with proton and electron radiations. Br. J. Radiol..

[37] Huang da W., Sherman B.T., Lempicki R.A. (2009). Systematic and integrative analysis of large gene lists using DAVID bioinformatics resources. Nat. Protoc..

[38] Barrett T., Wilhite S.E., Ledoux P., Evangelista C., Kim I.F., Tomashevsky M., Yefanov A., Marshall K.A., Phillippy K.H., Sherman P.M. (2013). NCBI GEO: Archive for functional genomics data sets-update. Nucleic Acids Res..

[39] Medhora M., Gao F., Fish B.L., Jacobs E.R., Moulder J.E., Szabo A. (2012). Dose-modifying factor for captopril for mitigation of radiation injury to normal lung. J. Radiat. Res..

[40] Barendsen G.W. (1982). Dose fractionation, dose rate and iso-effect relationships for normal tissue responses. Int. J. Radiat. Oncol. Biol. Phys..

[41] Brenner D.J., Sachs R.K., Peters L.J., Withers H.R., Hall E.J. (2012). We forget at our peril the lessons built into the α/β model. Int. J. Radiat. Oncol. Biol. Phys..

[42] Barazzuol L., Burnet N.G., Jena R., Jones B., Jefferies S.J., Kirkby N.F. (2010). A mathematical model of brain tumors response to radiotherapy and chemotherapy considering radiobiological aspects. J. Theor. Biol..

[43] Nieder C., Baumann M. (2011). Re-Irradiation: New Frontiers, Medical Radiology.

[44] Williams M.V., Denekamp J., Fowler J.F., Steel G.G. (1985). A review of a/b ratios for experimental tumours: Implications for clinical studies of altered fractionation. Basic Clinical Radiobiology.

[45] Bravata V., Minafra L., Russo G., Forte G.I., Cammarata F.P., Ripamonti M., Messa C., Casarino C., Augello G., Costantini F. (2015). High dose ionizing radiation regulates gene expression changes in MCF7 breast cancer cell Line. Anticancer Res..

[46] Johnson R., Halder G. (2014). The two faces of Hippo: Targeting the Hippo pathway for regenerative medicine and cancer treatment. Nat. Rev. Drug Discov..

[47] Orr B.A., Bai H., Odia Y., Jain D., Anders R.A., Eberhart C.G. (2011). Yes-associated protein 1 is widely expressed in human brain tumors and promotes glioblastoma growth. J. Neuropathol. Exp. Neurol..

[48] Yang R., Wu Y., Zou J., Zhou J., Wang M., Hao X., Cui H. (2016). The Hippo transducer TAZ promotes cell proliferation and tumor formation of glioblastoma cells through EGFR pathway. Oncotarget.

[49] Bae J.S., Kim S.M., Lee H. (2017). The Hippo signaling pathway provides novel anti-cancer drug targets. Oncotarget.

[50] Kim S., Jho E.H. (2016). Merlin, a regulator of Hippo signaling, regulates Wnt/β-catenin signaling. BMB Rep..

[51] Dong Z., Zhou L., Han N., Zhang M., Lyu X. (2015). Wnt/β-catenin pathway involvement in ionizing radiation-induced invasion of U87 glioblastoma cells. Strahlenther. Onkol..

[52] Wang H., Sun T., Hu J., Zhang R., Rao Y., Wang S., Bigner D.D., Chen R., McLendon R.E., Friedman A.H. (2014). miR-33a promotes glioma-initiating cell self-renewal via PKA and NOTCH pathways. J. Clin. Invest..

[53] Daniel P.M., Filiz G., Mantamadiotis T. (2016). Sensitivity of GBM cells to cAMP agonist-mediated apoptosis correlates with CD44 expression and agonist resistance with MAPK signaling. Cell Death Dis..

[54] Meyer R.G., Küpper J.H., Kandolf R., Rodemann H.P. (2002). Early growth response-1 gene (Egr-1) promoter induction by ionizing radiation in U87 malignant glioma cells in vitro. Eur. J. Biochem..

[55] Ghosh S., Paul A., Sen E. (2013). Tumor necrosis factor α-induced hypoxia-inducible factor 1α-β-catenin axis regulates major histocompatibility complex class I gene activation through chromatin remodeling. Mol. Cell Biol..

[56] Lino M.M., Merlo A. (2011). PI3Kinase signaling in glioblastoma. J. Neurooncol..

[57] Minafra L., Bravata V., Russo G., Forte G.I., Cammarata F.P., Ripamonti M., Messa C., Candiano G., Cervello M., Giallongo A. (2015). Gene expression profiling of MCF10A breast epithelial cells exposed to IOERT. Anticancer Res..

[58] Iozzo R.V., Sanderson R.D. (2011). Proteoglycans in cancer biology, tumour microenvironment and angiogenesis. J. Cell Mol. Med..

[59] Kazanskaya G.M., Tsidulko A.Y., Volkov A.M., Kiselev R.S., Suhovskih A.V., Kobozev V.V., Grigorieva E.V., Gaytan A.S., Aidagulova S.V., Krivoshapkin A.L. (2018). Heparan sulfate accumulation and perlecan/HSPG2 up-regulation in tumour tissue predict low relapse-free survival for patients with glioblastoma. Histochem. Cell Biol..

[60] Giatromanolaki A., Sivridis E., Mitrakas A., Kalamida D., Zois C.E., Haider S., Koukourakis M.I., Piperidou C., Pappa A., Gatter K.C. (2014). Autophagy and lysosomal related protein expression patterns in human glioblastoma. Cancer Biol. Ther..

[61] Yasui L.S., Duran M., Andorf C., Kroc T., Owens K., Allen-Durdan K., Becker R., Schuck A., Grayburn S. (2016). Autophagic flux in glioblastoma cells. Int. J. Radiat. Biol..

[62] Yaghi L., Poras I., Simoes R.T., Donadi E.A., Tost J., Daunay A., Moreau P., de Almeida B.S., Carosella E.D. (2016). Hypoxia inducible factor-1 mediates the expression of the immune checkpoint HLA-G in glioma cells through hypoxia response element located in exon 2. Oncotarget.

[63] Di Maggio F.M., Minafra L., Forte G.I., Cammarata F.P., Lio D., Messa C., Bravatà V., Gilardi M.C. (2015). Portrait of inflammatory response to ionizing radiation treatment. J. Inflamm..

[64] Kim K.W., Mutter R.W., Cao C., Albert J.M., Shinohara E.T., Sekhar K.R., Lu B. (2006). Inhibition of signal transducer and activator of transcription 3 activity results in down-regulation of Survivin following irradiation. Mol. Cancer Ther..

[65] Yu H., Pardoll D., Jove R. (2009). STATs in cancer inflammation and immunity: A leading role for STAT3. Nat. Rev. Cancer..

[66] Sun Y., Cheng M.K., Thomas R.L.G., Kilian M.J., Kai B., Kriajevska M., Manson M. (2013). Inhibition of STAT signalling in bladder cancer by diindolylmethane: Relevance to cell adhesion, migration and proliferation. Curr. Cancer Drug Targets..

[67] Du Y.C., Gu S., Zhou J., Wang T., Cai H., MacInnes M.A., Chen X., Bradbury E.M. (2006). The dynamic alterations of H2AX complex during DNA repair detected by a proteomic approach reveal the critical roles of Ca(2+)/calmodulin in the ionizing radiationinduced cell cycle arrest. Mol. Cell. Proteom..

[68] Lao Y., Chang D.C. (2008). Mobilization of Ca2+ from endoplasmic reticulum to mitochondria plays a positive role in the early stage of UV-or TNFα-induced apoptosis. Biochem. Biophys. Res. Commun..

[69] Combs S.E., Bohl J., Elsässer T., Weber K.J., Schulz-Ertner D., Debus J., Weyrather W.K. (2009). Radiobiological evaluation and correlation with the local effect model (LEM) of carbon ion radiation therapy and temozolomide in glioblastoma cell lines. Int. J. Radiat. Biol..

[70] Combs S.E., Zipp L., Rieken S., Habermehl D., Brons S., Winter M., Weber K.J., Haberer T., Debus J. (2012). In vitro evaluation of photon and carbon ion radiotherapy in combination with chemotherapy in glioblastoma cells. Radiat. Oncol..

[71] Barazzuol L., Jena R., Burnet N.G., Jeynes J.C., Merchant M.J., Kirkby K.J., Kirkby N.F. (2012). In Vitro Evaluation of Combined Temozolomide and Radiotherapy Using X Rays and High-Linear Energy Transfer Radiation for Glioblastoma. Radiat. Res..

[72] Cirrone G.A.P., Cuttone G., Lojacono P.A., Lo Nigro S., Mongelli V., Patti I.V., Privitera G., Raffaele L., Rifuggiato D., Sabini M.G. (2004). A 62-MeV proton beam for the treatment of ocular melanoma at laboratori nazionali del sud-INFN. IEEE Transact. Nuclear Sci..

[73] Cirrone G.A.P., Cuttone G., Lo Nigro S., Mongelli V., Raffaele L., Sabini M.G. (2006). Dosimetric characterization of CVD diamonds in photon, electron and proton beams. Nuclear Physics B (Proc. Suppl.).

[74] Sartini L., Simeone F., Pani P., Lo Bue N., Marinaro G., Grubich A., Gasparoni F., Lobko A., Etiope G., Gapone A. (2017). Nuclear Instruments and Methods in Physics Research Section A: Accelerators, Spectrometers, Detectors and Associated Equipment. Nucl. Instrum. Methods Phys. Res. A.

[75] Militello C., Rundo L., Conti V., Minafra L., Cammarata F.P., Mauri G., Porcino N., Gilardi M.C. (2017). Area-based cell colony surviving fraction evaluation: A novel fully automatic approach using general-purpose acquisition hardware. Comput. Biol. Med..

[76] Minafra L., Porcino N., Bravatà V., Gaglio D., Bonanomi M., Amore E., Baglio M., Cammarata F.P., Russo G., Militello C. (2019). Radiosensitizing effect of curcumin-loaded lipid nanoparticles in breast cancer cells. Sci. Rep..

