# Supplementary material for: Proton Therapy and Src Family Kinase Inhibitor Combined Treatments on U87 Human Glioblastoma Multiforme Cell Line
Source: Int J Mol Sci. 2019 Sep 24;20(19):4745. doi: 10.3390/ijms20194745 (PMC6801826; doi:10.3390/ijms20194745)
Supplement: Supplementary file 1 [file ijms-20-04745-s001.pdf]

Table S1. Pubmatrix analysis

	Cancer	Glioblastoma	Radiation	SRC	Ionizing Radiation	Apoptosis	Inflammation	DNA repair	DNA damage	Radioresistance
**SRC**	12616	201	647	35709	189	3226	1314	124	271	19
**STAT3**	10679	410	599	1139	135	5138	3756	146	314	69
**CMYC**	456	6	21	7	3	171	16	6	16	2
**CCND1**	3607	52	141	46	33	1027	109	96	121	9
**PI3K**	19176	709	1215	1614	317	10820	2866	471	762	140
**PDK1**	737	29	38	62	11	389	80	9	21	4
**AKT**	37969	1358	2617	2886	783	26010	5800	756	1858	279
**BAD**	7645	93	970	86	137	3893	1352	121	411	19
**MTOR**	16216	517	959	344	202	6657	1576	340	663	77
**KRAS**	11422	52	475	71	46	918	302	459	179	12
**FOS**	5042	55	1081	549	315	1703	1381	204	458	6
**PXN**	351	6	16	195	4	75	28	2	8	4
